# Spectral Reflectance as a Unique Tissue Identifier in Healthy Humans and Inhalation Injury Subjects

**DOI:** 10.3390/s22093377

**Published:** 2022-04-28

**Authors:** Carlos N. Bedolla, Catherine Rauschendorfer, Drew B. Havard, Blaine A. Guenther, Julie A. Rizzo, August N. Blackburn, Kathy L. Ryan, Megan B. Blackburn

**Affiliations:** 1 U.S. Army Institute of Surgical Research, JBSA Fort Sam Houston, San Antonio, TX 78234, USA; carlosn.bedolla@gmail.com (C.N.B.); catherine.rauschendorfer.civ@mail.mil (C.R.); julie.a.rizzo.mil@mail.mil (J.A.R.); meganbblackburn@gmail.com (M.B.B.); 2 Naval Medical Research Unit San Antonio, JBSA Fort Sam Houston, San Antonio, TX 78234, USA; drew.b.havard.mil@mail.mil; 3 59th Medical Wing, Brooke Army Medical Center, JBSA Fort Sam Houston, San Antonio, TX 78234, USA; blaine.a.guenther.mil@mail.mil; 4 Blackburn Statistics, LLC, San Antonio, TX 78260, USA; august.blackburn.phd@gmail.com

**Keywords:** spectral reflectance, tracheal intubation, inhalation injury, airway management

## Abstract

Tracheal intubation is the preferred method of airway management, a common emergency trauma medicine problem. Currently, methods for confirming tracheal tube placement are lacking, and we propose a novel technology, spectral reflectance, which may be incorporated into the tracheal tube for verification of placement. Previous work demonstrated a unique spectral profile in the trachea, which allowed differentiation from esophageal tissue in ex vivo swine, in vivo swine, and human cadavers. The goal of this study is to determine if spectral reflectance can differentiate between trachea and other airway tissues in living humans and whether the unique tracheal spectral profile persists in the presence of an inhalation injury. Reflectance spectra were captured using a custom fiber-optic probe from the buccal mucosa, posterior oropharynx, and trachea of healthy humans intubated for third molar extraction and from the trachea of patients admitted to a burn intensive care unit with and without inhalation injury. Using ratio comparisons, we found that the tracheal spectral profile was significantly different from buccal mucosa or posterior oropharynx, but the area under the curve values are not high enough to be used clinically. In addition, inhalation injury did not significantly alter the spectral reflectance of the trachea. Further studies are needed to determine the utility of this technology in a clinical setting and to develop an algorithm for tissue differentiation.

## 1. Introduction

Tracheal intubation is a common life-saving procedure performed in pre-hospital and emergency settings when a patient has difficulty breathing or is unable to protect their airway. This procedure can be difficult and is often performed under sub-optimal conditions, which can lead to patient complications due to inadvertent esophageal intubation [1,2,3]. Furthermore, multiple attempts at intubation significantly increase the risk to the patient [2]. Proper placement can be confirmed through direct visualization of the tube passing through the vocal cords and observation of the chest rising and falling with ventilation, capnography, or capnometry. The use of end-tidal carbon dioxide monitoring in pre-hospital environments allows for earlier detection of esophageal intubation [4,5,6,7]. However, end-tidal carbon dioxide-monitoring devices are associated with a high rate of false positives due to previous mouth-to-mouth resuscitation, exposure to acidic contents, or drugs [8,9,10,11]. Given this, a novel solution to improve emergency tracheal intubation placement is a priority. One such solution would be to develop an automated mechanism whereby a pre-hospital provider could receive continuous feedback during the intubation process itself to ensure that the tracheal tube is properly placed in the trachea rather than the esophagus. 

Our previous work using ex vivo swine tissue, in vivo swine, and human cadavers showed that tracheal and esophageal tissue reflect white light in distinct ways [12,13]. The differences in the layers of tissue affect light scattering properties, including the rigid geometry and hyaline cartilage make-up of the trachea. Conversely, the esophagus has a folded, collapsed geometry, and the underlying muscularis presents a much stronger absorption layer [14,15]. In previous studies, we demonstrated the existence of a unique spectral profile in tracheal tissue, a peak at 561nm and troughs at 543 nm and 578 nm, which differentiates tracheal from esophageal tissue. It is currently unknown whether the tracheal spectral profile persists in live human tissue, how other upper airway tissues may reflect white light, or how tracheal injury may impact the unique spectral profile.

Determining the effectiveness of spectral reflectance in living humans is vital to establishing the clinical utility of the technology. In addition, given that this technology would be used in a far-forward setting, its usefulness within an injured airway, such as inhalation injury, must be further investigated. Burns are a significant mechanism of injury on the battlefield and in civilian settings, and inhalation injury occurs in approximately 10–20% of burn patients [16]. Because inhalation injury results in airway edema and carbon deposits, it is unknown how it will affect tracheal spectral reflectance properties. In previous ex vivo work, however, we demonstrated that the presence of soot did not alter the tracheal spectral reflectance properties [17], but this finding awaits confirmation in actual patients with inhalation injury.

This study aimed to determine if spectral reflectance can differentiate between trachea and other airway tissues in healthy humans and whether the unique tracheal spectral profile persists in the presence of an inhalation injury. If the unique spectral signature of the trachea found in other species and human cadavers persists, it is possible that this information could be used in future devices designed to facilitate rapid and correct placement of an endotracheal tube. This study was designed to compare the spectral reflectance of trachea with other tissues, including buccal mucosa and posterior oropharynx, in healthy subjects and tracheal reflectance only in burn patients with and without inhalation injury. We hypothesized that the trachea would exhibit the unique reflectance spectra signature previously documented and that this would differ from the spectra exhibited by buccal mucosa and posterior oropharynx. Furthermore, we hypothesized that the presence of inhalation injury would not affect the unique reflectance spectra signature of the trachea.

## 2. Materials and Methods

### 2.1. Spectral Reflectance Measurements

**Figure 1:**
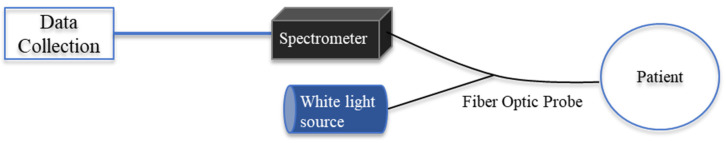
Diagram of spectral probe experimental setup.

For all subjects, reflectance spectra were captured using a custom-designed fiber-optic reflection probe (Gulf Photonics, Oldsmar, FL, USA). The fiber optic probe has a 200-μm core, 2-m long bifurcated reflection probe with a central light-emitting fiber with 6 collection fibers surrounding it to capture the light reflected from the luminal tissue. The probe was connected to a spectrometer (Ocean Optics, Orlando, FL, USA) and white light source and connected via USB to a laptop with the OceanView software (Ocean Optics, Orlando, FL, USA), as shown in Figure 1. The probe was calibrated prior to testing by collecting dark and white reference spectra. To capture the white spectrum, the white light was turned on, and the end of the probe was inserted into a white integration sphere (FIOS-1, Ocean Optics, Largo, FL, USA). The dark spectrum was collected by turning off the white light source and covering the end of the probe with sterile black fabric. 

#### 2.1.1. Healthy Human Subjects

Healthy human subjects were recruited from the Oral Surgery Department at Brooke Army Medical Center. As part of a weekly training program, the department performs third molar extractions as an outpatient procedure wherein the patient is placed under general anesthesia and intubated prior to the procedure. The inclusion criteria for healthy human subjects (n = 16) were as follows: age between 18–60 years, intubated as standard of care for the oral surgical procedure, and consent provided prior to data collection. Subjects were excluded if they were outside the age range or had a history of asthma or other tracheal disease.

Following complete extraction of the molars but before extubation, the Oral Maxillofacial Surgeon Investigator (DH, BG) inserted a nasopharyngeal airway (NPA) through the nasal cavity. The sterilized fiber-optic probe was then inserted one inch beyond the end of the NPA to capture spectral data from the posterior oropharynx. The probe was removed from the NPA and placed in the patient’s mouth against the buccal mucosa to collect spectral data from the cheek. Lastly, the tracheal tube was unhooked from the ventilation circuit, and the spectral probe was inserted through the tracheal tube until the distal end of the probe was one inch beyond the end of the tube to collect spectral data from the trachea. A total of three reflectance captures were collected from each tissue group.

#### 2.1.2. Burn Intensive Care Unit (BICU) Subjects

Burn subjects were between the ages of 18 and 60, admitted to the BICU at the U.S. Army Institute of Surgical Research, who were intubated and underwent bronchoscopy as part of the standard of care to assess the severity and extent of any inhalation injuries present.

Spectral reflectance captures were collected within eight hours of the initial standard of care bronchoscopy. A bronchoscope adapter was placed on the tracheal tube so the subject could remain on the ventilator. The sterilized fiber-optic probe was inserted into the tracheal tube through the adapter until the distal end of the probe was approximately one inch beyond the end of the tube into the tracheal lumen to capture spectral reflectance of the light reflected off the tissue. A total of three spectral captures were taken and saved to the designated computer file. The Burn Surgeon investigator (JR) assessed inhalation injury grade from photographic images of the trachea using the abbreviated injury score (AIS) and pathological grade. Images were graded in batches as they became available.

### 2.2. Data Analysis

Demographic data for BICU subjects with inhalation injury and BICU subjects without inhalation injury were analyzed using an unpaired, two-tailed t-test with Welch’s correction given the assumption of unequal standard deviations.

**Figure 2:**
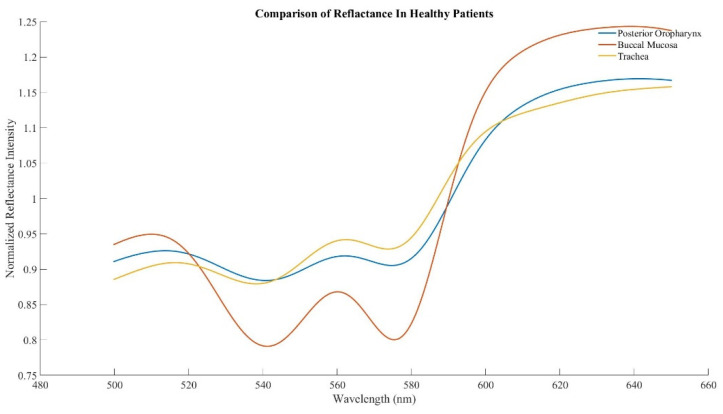
Averaged spectra in healthy human subjects acquired from the buccal mucosa, posterior oropharynx, and trachea.

For the purpose of visualization, in Figures 2 and 4, reflectance data were imported into MATLAB, and all spectra were cropped to focus on the 500 nm to 650 nm range, as we have described previously [13]. The reflectance spectra for buccal mucosa, posterior oropharynx, and/or trachea were averaged across samples by summing the value at each data point across the captured profiles and dividing by the number of profiles to arrive at one reflectance spectrum representing each tissue type (buccal mucosa, posterior oropharynx, trachea). These mean values were smoothed using convolution as described previously [13]. The averaged and smoothed reflectance spectra were plotted by tissue type for qualitative comparison. The tracheal signature of interest is characterized by a “hump” over the range from 543 nm to 578 nm. Ratio B was termed based on 543 nm being approximately blue light and Ratio Y for being approximately yellow light on the visual spectrum [1]. Target wavelengths of 543 nm, 561 nm, and 578 nm were chosen based on commercially available glass filters to represent a detection algorithm that could be implemented by developed prototypes and because these wavelengths capture the relative prominence of the “hump”.

For the purpose of statistical analysis, each reflectance spectra was normalized, and smoothed reflectance values were extracted using kernel regression as previously described [2]. Briefly, the reflectance values were divided by the sum of all values across the 500 nm to 650 nm range. This has the effect of normalizing the mean value of each reflectance spectra to 1 (which makes visually comparing individual spectra to each other easier) and maintains the relative spectral reflectance values within spectra proportionally with respect to each other. We then used a Gaussian kernel to perform kernel regression, implemented within the np package in R [18]. Smoothed reflectance wavelengths of 543, 561, and 578 were interpolated from the smoothed reflectance spectra. Ratio B (561/543) and Ratio Y (561/578) values, the relative comparisons between wavelengths of interest, were calculated for each spectrum capture using the interpolated values. The values for Ratio B and Ratio Y are equivalent to the same ratios calculated if the data were not normalized, which would be advantageous for the simplicity of calculating the ratios if a device were developed using commercially available filters. 

Ratio B and Ratio Y were modeled using linear mixed models. The spectral reflectance ratios were treated as the dependent variable. Individuals were treated as random effects to account for non-independence between multiple measures in the same person. Differences between groups were treated as fixed effects. The area under the curve (AUC) for receiver operator characteristic (ROC) curves was calculated using the R package pROC [19].

## 3. Results

A total of 38 subjects were enrolled in the study: 16 healthy, 13 admitted to the BICU with inhalation injury, and 9 admitted to the BICU with no inhalation injury. Demographics for all subjects are shown in Table 1.

**Figure 3:**
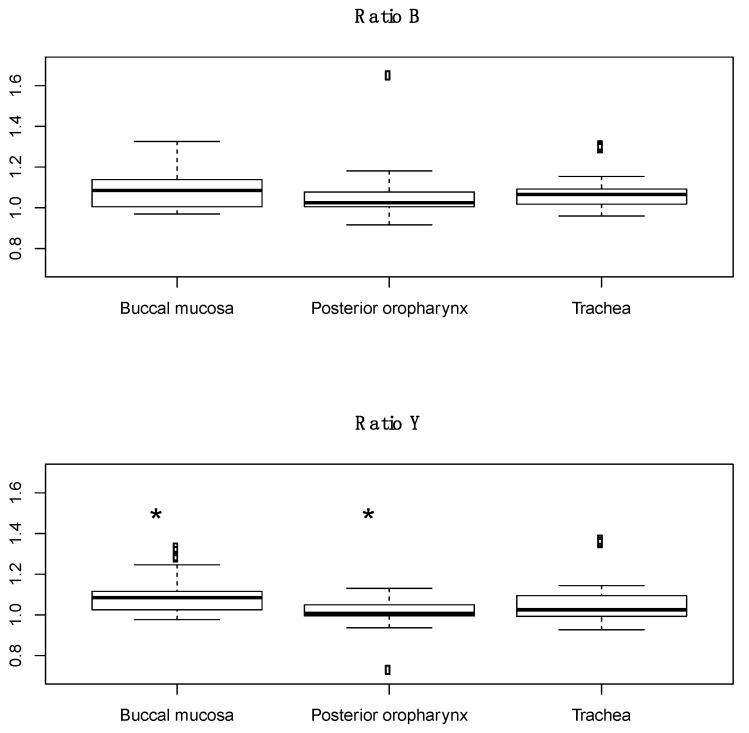
Boxplots of ratio B and ratio Y for buccal mucosa, posterior oropharynx, and tracheal tissues from healthy human subjects. Dots in the boxplots are outliers, defined as being 1.5 times the interquartile range beyond the first and third quartiles. * *p* < 0.01 compared to trachea.

Spectral scans were taken from the buccal mucosa, posterior oropharynx, and trachea in healthy subjects, with a total of 48 distinct scans per tissue (3 scans per tissue in 16 subjects). The previously identified characteristic peak at 561 nm and adjacent troughs at 543 nm and 578 nm were present in all tissue types (Figure 2). Ratio B did not differentiate tissue types, whereas the average ratio Y value was significantly different in each of three possible pairwise comparisons between the three tissues (*p* < 0.01, Figure 3). The AUC for ROC curves for comparisons of trachea vs. buccal mucosa and trachea vs. posterior oropharynx were 0.6654 and 0.6181, respectively.

**Figure 4:**
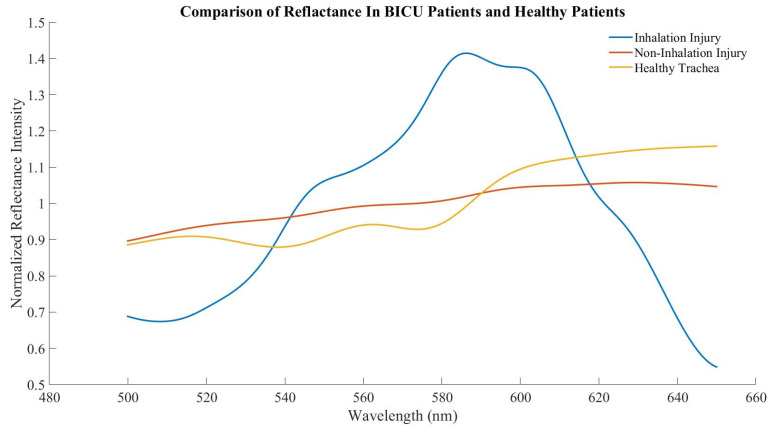
Averaged tracheal spectra from healthy human subjects, BICU subjects with inhalation injury, and BICU subjects without inhalation injury.

**Figure 5:**
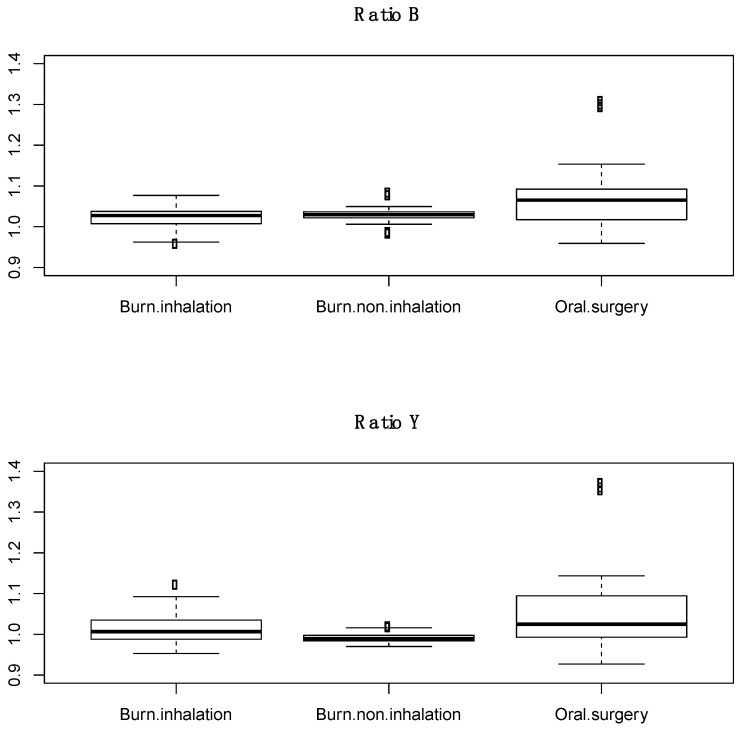
Boxplots of ratio B and ratio Y of trachea from burn subjects with inhalation injury, burn subjects without inhalation injury, and healthy human subjects. Dots in the boxplots are outliers, defined as being 1.5 times the interquartile range beyond the first and third quartiles.

Subjects with inhalation injury were less likely to be male and had greater body mass index as compared to subjects with no inhalation injury. There were no significant differences between subjects in age, % total body surface area burn, or 24 h resuscitation volumes. The average inhalation injury score was 1.9 ± 0.9. Scores were calculated from an average of 3.4 images per subject (range 2–6). A total of 39 and 27 spectral scans were taken from the trachea in BICU patients with and without inhalation injury, respectively. The expected tracheal spectral profile was largely absent in both groups (Figure 4). Additionally, there was no significant difference in ratio B or ratio Y between those with and without inhalation injury (Figure 5).

## 4. Discussion

The goals of this study were threefold, to determine whether (1) the unique tracheal spectral profile persists in live humans; (2) other upper airway tissues can be differentiated from trachea; and (3) inhalation injury significantly impacts how tracheal tissue reflects white light. To that end, we found that the previously established tracheal profile, with troughs at 543 nm and 578 nm and a peak at 561 nm, was present in healthy human subjects. We had previously determined that spectral reflectance could differentiate tracheal and esophageal tissues, and ratio Y of buccal mucosa and posterior oropharynx was statistically different from trachea, while ratio B was similar. Lastly, burn subjects with and without inhalation injury have a qualitatively different tracheal profile but no significant difference in ratios B or Y.

Previously, our team showed that spectral reflectance could differentiate between esophageal and tracheal tissues in ex vivo swine, in vivo swine even during hypoxia, and human cadavers [12,13,20]. In all instances, there was a statistically significant difference in ratios B and Y between the tissue types, which we hypothesized could be used as a means to validate tracheal tube placement if the technology was built directly into the tube. Furthermore, we confirmed that this difference persisted in the presence of saline, blood, vomit, or soot in the trachea in an initial attempt to simulate injury [17]. Herein, we hypothesized that not only would the tracheal spectral profile be present in live human tissue but that the trachea would continue to differ from other upper airway tissues, similar to the esophagus. Qualitative assessment confirmed that live human tracheal tissue does, in fact, produce a tracheal spectral profile similar to previous studies. However, the mean for ratio B was not statistically different between tissues. The mean for ratio Y was statistically significant, but the AUC of the ROC curves is not high enough to be used clinically. We had previously hypothesized that the unique tracheal profile was due to its tissue makeup and high level of oxygenated hemoglobin [13]. However, studies have shown that other tissues, such as skin, produce a similar spectral profile [21], which may explain the similar profile seen in buccal mucosa and posterior oropharynx tissues. In addition, a previous study from 2018 showed that near-infrared spectroscopy could be used to differentiate tracheal locations, which the authors hypothesized was due to differences in protein and water content [22]. In our studies, we have not seen a difference across tracheal location, but location has not been controlled for, though future studies are needed to make a clear determination.

We were unable to collect spectral data from the esophagus of healthy humans due to the spectral probe size and desire to minimize the invasiveness of experimental procedures, i.e., we could not easily and safely pass the probe into the esophagus in these patients. A previous study examined the spectral reflectance of nasal mucosa in living subjects. While they examined the entirety of the visual spectrum, they did report a peak at 560 nm, similar to our findings [23]. However, this is the first study to our knowledge to investigate how other upper airway tissues reflect white light, and an additional study will be needed to verify that esophageal tissue in live humans can be differentiated from these tissues. This will be necessary for the technology to progress into a form that is useful for far-forward providers, such as incorporating it directly into the tracheal tube or tip of a bougie, with automated algorithms that provide real-time decision-support to the provider.

Inhalation injury complicates burns in approximately 10–20% of patients and increases morbidity and mortality. Inhalation injury diagnosis remains subjective, and currently, inhalation injuries are graded based on clinical examination and bronchoscope image analysis [15]. Several modalities can be used for inhalation injury confirmation, including bronchoscopy, chest computed tomography, carboxyhemoglobin measurement, radionuclide imagining with Xenon, and pulmonary function testing. However, these tend to lack sensitivity, and interpretation remains largely subjective. Given these limitations, a quantitative measure of inhalation injury severity would significantly improve diagnosis [16]. With that in mind, we hypothesized that spectral reflectance might offer such a quantitative measure if the tracheal spectral profile was, in fact, affected by inhalation injury. To our surprise, the unique tracheal profile was largely absent in all BICU subjects, including those without inhalation injury, and we did not find any significant difference between tracheal spectral reflectance in subjects with or without inhalation injury. It is currently unknown how the downstream effects of burn injury and resuscitation [24] may impact spectral reflectance even in cases without inhalation injury. A caveat to these findings is the relatively small n, especially in regards to burn subjects without inhalation injury and those with more severe inhalation injuries. To fully determine the quantitative value of spectral reflectance, a larger study with a greater number of severe inhalation injuries is needed.

There are limitations to this study. Given the size of the spectral reflectance probe, it was placed in the trachea and NPA individually; therefore, we did not have the capability to verify the specific location of the probe tip. Any future studies would need to have simultaneous images taken to confirm the exact location. In this way, it would be verified that the light is shining on the tissue and is not being impacted by the tracheal tube itself. Additionally, the number of subjects, specifically in regards to inhalation injury and non-inhalation injury, was relatively low, as entry of patients into the BICU decreased during the COVID pandemic.

In conclusion, tracheal tissue in healthy humans exhibits the expected spectral profile, and while ratio Y was significantly different between other tissue types, the AUC was not clinically relevant. In addition, the tracheal spectral profile in burn-injured patients with and without inhalation injury was not different, which indicates possible utility for spectral reflectance to verify tracheal placement in burn-injured patients but makes it unlikely that the technology could be used for quantitative diagnosis of injury.

## Figures and Tables

**Table 1:** Subject Demographics.

Characteristic	Healthy (n = 16)	Inhalation Injury (n = 13)	Non-Inhalation Injury (n = 9)	*p*-Value
Male (%)	11 (68)	8 (62) *	9 (100)	0.018
Age, years	23.1 ± 4.2	38.8 ± 12.9	45.5 ± 12.5	0.238
BMI	27.3 ± 3.8	33.5 ± 7.3 *	27.2 ± 4.3	0.023
TBSA, %	N/A	29.6 ± 26.4	25.1 ± 22.4	0.672
Resuscitation (mL)	N/A	14877 ± 8725	11270 ± 7210	0.303

Values are mean ± SD, * *p* < 0.05 vs. Non-Inhalation Injury. Resuscitation indicates the total amount of resuscitation fluid infused over a 24 h period. BMI, body mass index; TBSA, total body surface area.

## Data Availability

Not applicable.

## References

[1] Cobas M.A., De la Peña M.A., Manning R., Candiotti K., Varon A.J. (2009). Prehospital Intubations and Mortality: A Level 1 Trauma Center Perspective. Anesthesia Analg..

[2] Sakles J.C., Chiu S., Mosier J., Walker C., Stolz U. (2013). The Importance of First Pass Success When Performing Orotracheal Intubation in the Emergency Department. Acad. Emerg. Med..

[3] Hasegawa K., Shigemitsu K., Hagiwara Y., Chiba T., Watase H., Brown C.A., Brown D.F., Japanese Emergency Medicine Research Alliance Investigators (2012). Association between Repeated Intubation Attempts and Adverse Events in Emergency Departments: An Analysis of a Multicenter Prospective Observational Study. Ann. Emerg. Med..

[4] Silvestri S., Ralls G.A., Krauss B., Thundiyil J., Rothrock S.G., Senn A., Carter E., Falk J. (2005). The Effectiveness of Out-of-Hospital Use of Continuous End-Tidal Carbon Dioxide Monitoring on the Rate of Unrecognized Misplaced Intubation Within a Regional Emergency Medical Services System. Ann. Emerg. Med..

[5] Lockey D., Crewdson K., Weaver A., Davies G. (2014). Observational study of the success rates of intubation and failed intubation airway rescue techniques in 7256 attempted intubations of trauma patients by pre-hospital physicians. Br. J. Anaesth..

[6] Jooste R., Roberts F., Mndolo S., Mabedi D., Chikumbanje S., Whitaker D.K., O’Sullivan E.P. (2019). Global Capnography Project (GCAP): Implementation of capnography in Malawi—An international anaesthesia quality improvement project. Anaesthesia.

[7] Cook T.M. (2018). Strategies for the prevention of airway complications—A narrative review. Anaesthesia.

[8] Keller M.W., Han P.P., Galarneau M.R., Brigger M.T. (2015). Airway Management in Severe Combat Maxillofacial Trauma. Otolaryngol. Neck Surg..

[9] Hughes K.E., Biffar D., Ahanonu E.O., Cahir T.M., Hamilton A., Sakles J.C. (2018). Evaluation of an Innovative Bleeding Cricothyrotomy Model. Cureus.

[10] Neumar R.W., Otto C.W., Link M.S., Kronick S.L., Shuster M., Callaway C.W., Kudenchuk P.J., Ornato J.P., McNally B., Silvers S.M. (2010). Part 8: Adult Advanced Cardiovascular Life Support: 2010 American Heart Association guidelines for cardiopulmonary resuscitation and emergency cardiovascular care. Circulation.

[11] Rabitsch W., Nikolic A., Schellongowski P., Kofler J., Kraft P., Krenn C.G., Staudinger T., Locker G.J., Knöbl P., Hofbauer R. (2004). Evaluation of an end-tidal portable ETCO_2_ colorimetric breath indicator (COLIBRI). Am. J. Emerg. Med..

[12] Nawn C.D., Souhan B.E., Carter R., Kneapler C., Fell N., Ye J.Y. (2016). Distinguishing tracheal and esophageal tissues with hyperspectral imaging and fiber-optic sensing. J. Biomed. Opt..

[13] Nawn C.D., Blackburn M.B., De Lorenzo R.A., Ryan K.L. (2019). Using spectral reflectance to distinguish between tracheal and oesophageal tissue: Applications for airway management. Anaesthesia.

[14] Fuchs J.R., A Nasseri B., Vacanti J.P. (2001). Tissue engineering: A 21st century solution to surgical reconstruction. Ann. Thorac. Surg..

[15] Pitris C., Brezinski M.E., Bouma B.E., Tearney G.J., Southern J.F., Fujimoto J.G. (1998). High Resolution Imaging of the Upper Respiratory Tract with Optical Coherence Tomography: A feasibility study. Am. J. Respir. Crit. Care Med..

[16] Walker P.F., Buehner M.F., Wood L.A., Boyer N.L., Driscoll I.R., Lundy J.B., Cancio L.C., Chung K.K. (2015). Diagnosis and management of inhalation injury: An updated review. Crit. Care.

[17] Berard D., Sen C., Nawn C., Blackburn A., Ryan K., Blackburn M. (2020). Spectral Reflectance Can Differentiate Tracheal and Esophageal Tissue in the Presence of Bodily Fluids and Soot. Sensors.

[18] Hayfield T., Racine J.S. (2008). Nonparametric econometrics: The Np package. J. Stat. Softw..

[19] Robin X., Turck N., Hainard A., Tiberti N., Lisacek F., Sanchez J.-C., Müller M. (2011). pROC: An open-source package for R and S+ to analyze and compare ROC curves. BMC Bioinform..

[20] Blackburn M.B., Nawn C.D., Ryan K.L. (2019). Testing of novel spectral device sensor in swine model of airway obstruction. Physiol. Rep..

[21] Angelopoulou E. (1999). The Reflectance Spectrum of Human Skin.

[22] Linkov G., Hanifi A., Yousefi F., Tint D., Bolla S., Marchetti N., Soliman A.M.S., Pleshko N. (2018). Compositional Assessment of Human Tracheal Cartilage by Infrared Spectroscopy. Otolaryngol. Neck Surg..

[23] Ohyama M., Furuta S., Kurono Y., Yano H., Ogawa K., Katsuda K. (1982). Reflectance spectrophotometric studies on mucosal pathology of the upper airway. Laryngoscope.

[24] Rae L., Fidler P., Gibran N. (2016). The Physiologic Basis of Burn Shock and the Need for Aggressive Fluid Resuscitation. Crit. Care Clin..

